# Transcriptomic profiling and bioinformatic insights into myocardial injury following aneurysmal subarachnoid hemorrhage

**DOI:** 10.3389/fneur.2025.1492398

**Published:** 2025-06-24

**Authors:** Zuoli Wu, Wenbo He, Weihao Ye, Shang Xu, Shengwei Wei, Baozi Huang, Pingping Li, Yanyan Tang, Chao Qin, Ying Liu, Ziming Ye

**Affiliations:** ^1^Department of Neurology, The First Affiliated Hospital of Guangxi Medical University, Nanning, China; ^2^Jiangbing Hospital, Guangxi Zhuang Autonomous Region, Nanning, Guangxi, China; ^3^Department of Rehabilitation Medicine, The Second Affiliated Hospital of Guangxi Medical University, Nanning, China

**Keywords:** aSAH-MI, lncRNA, miRNA, mRNA, ceRNA

## Abstract

**Background:**

Myocardial injury is a common complication of aneurysmal subarachnoid hemorrhage (aSAH) and is associated with poor outcomes. While RNA plays a critical role in pathophysiological processes, its expression patterns and functions in myocardial injury after aSAH (aSAH-MI) remain poorly understood.

**Objective:**

To construct the RNA expression profile of aSAH-MI patients, explore their biological functions, and establish a gene expression regulatory network for aSAH-MI. These findings provide a theoretical basis for understanding the RNA-level mechanisms underlying aSAH-MI.

**Methods:**

This study included 12 patients, comprising 6 aSAH-MI patients and 6 aSAH-nonMI patients (aSAH patients without myocardial injury). RNA sequencing was performed on three patients from each group to construct an RNA expression matrix. Differentially expressed genes (lncRNAs, miRNAs, mRNAs) were identified using the limma package in R. Gene Ontology (GO) and Kyoto Encyclopedia of Genes and Genomes (KEGG) analyses were performed. miRNA, lncRNA, and mRNA interactions were predicted using miRanda and RNAhybrid. An lncRNA-miRNA-mRNA interaction network was constructed with Cytoscape, and qRT-PCR validated selected genes in an additional six patients.

**Results:**

In aSAH-MI patients, 617 lncRNAs, 20 miRNAs, and 510 mRNAs were significantly differentially expressed, with 258, 13, and 244 being upregulated, and 359, 7, and 266 being downregulated, respectively (*P* < 0.05). Bioinformatic analysis revealed that the differentially expressed mRNAs were involved in biological processes such as ion transport, immune regulation, and myocardial contraction, and were associated with pathways related to vasodilation, nerve conduction, and cardiac function regulation. ceRNA analysis identified hsa-miR-4707-3p and hsa-miR-25-5p as potential network hubs. The lncRNAs with the highest connectivity were CELSR1-204, SLCO2B1-212, AEN-204, PPFIA4-205, and MIAT-219, while the mRNAs with the highest connectivity were CHI3L1, ADORA2A, PAX8, VWA3B, and KCNE1. These findings suggest these differentially expressed genes may serve as key regulators in mediating aSAH-MI. Validation through qRT-PCR in an additional cohort of six subjects confirmed the differential expression of selected genes.

**Conclusions:**

This study successfully constructed the RNA expression profiles in the blood of patients with aSAH-MI through transcriptome sequencing, identifying significant differentially expressed miRNAs, mRNAs, and lncRNAs. Bioinformatic analysis suggests these genes may play critical roles in the pathogenesis of aSAH-MI.

## 1 Introduction

Subarachnoid hemorrhage (SAH) is a life-threatening neurological emergency, with aneurysmal subarachnoid hemorrhage (aSAH) accounting for approximately 85% of spontaneous cases due to the rupture of intracranial aneurysms (1–3). Numerous studies have identified advanced age, elevated clinical severity scores, wide-necked aneurysms, and cardiopulmonary complications as independent predictors of poor outcomes in aSAH patients (4). Among these prognostic factors, cardiac complications are particularly critical yet often underrecognized (5). This is largely because such complications frequently present with subtle or non-specific symptoms, which may be overshadowed by more severe neurological impairments. Despite this, the incidence of cardiac complications—including electrocardiographic abnormalities, myocardial injury, and cardiac dysfunction—is notably high in aSAH patients and is closely associated with adverse outcomes.

Myocardial injury is defined by elevated cardiac troponin (cTn) levels exceeding the 99th percentile upper reference limit specific to the assay used (6). Myocardial injury following aSAH (aSAH-MI) represents a common manifestation of neurocardiac syndrome, with 20%−40% of aSAH patients exhibiting elevated cTn levels (7). This elevation is associated with increased clinical severity, higher rates of neurological complications, greater mortality, and poorer overall outcomes. Despite advances in secondary prevention, the incidence of post-stroke cardiac events remains significant, highlighting the urgent need to elucidate the mechanisms underlying aSAH-MI.

Advances in genomics have underscored the importance of the relationship between genes and disease. The majority of the human transcriptome consists of non-coding RNA (ncRNA), which, despite not encoding proteins, plays critical roles in various biological processes (8). Non-coding RNAs are classified based on length into short (< 200 nucleotides) and long (>200 nucleotides) categories, including microRNAs (miRNAs) and long non-coding RNAs (lncRNAs) (9, 10). These ncRNAs are essential for maintaining cellular homeostasis and have been implicated in a wide range of diseases. They are also thought to act as mediators of inter-organ and inter-cellular communication, although their roles in neurocardiac syndromes like aSAH-MI remain poorly understood.

Competing endogenous RNA (ceRNA) refers to RNA molecules that participate in complex transcriptional regulation networks by binding to microRNA response elements, thereby modulating gene expression (11). The analysis of ceRNA networks provides a novel framework for understanding RNA interactions and their regulatory functions in gene expression. Transcriptome sequencing (RNA-Seq) offers a comprehensive approach to analyze differentially expressed mRNAs and ncRNAs, facilitating the construction of ceRNA networks that can illuminate gene regulatory mechanisms in disease states. However, studies employing RNA-Seq to investigate aSAH-MI are currently limited.

This study aims to construct the RNA expression profile of aSAH-MI patients, focusing on lncRNAs, miRNAs, and mRNAs. By utilizing RNA sequencing, we will develop an RNA expression matrix specific to myocardial injury following aSAH. The study will identify significantly differentially expressed genes and conduct extensive bioinformatics analyses, including pathway, functional, and ceRNA network analyses, to establish a comprehensive gene expression regulatory network for aSAH-MI.

## 2 Materials and methods

### 2.1 Patients and specimens

This study involved the collection of peripheral blood samples from 12 patients diagnosed with aneurysmal subarachnoid hemorrhage (aSAH), of which 6 patients developed secondary myocardial injury (aSAH-MI group), while the remaining 6 patients did not exhibit myocardial injury (aSAH-nonMI group). The diagnosis of myocardial injury secondary to aSAH was based on the criteria established by the American Heart Association/American Stroke Association (AHA/ASA) (12, 13). All participants were inpatients at the First Affiliated Hospital of Guangxi Medical University from October 2022 to October 2023. Informed consent was obtained from all participants or their legal representatives in compliance with the ethical standards outlined in the Declaration of Helsinki. Exclusion criteria included a history of heart disease, a history of cancer, severe renal or hepatic dysfunction, or other significant comorbid conditions. Four milliliters of fresh peripheral blood samples were collected using EDTA tubes, immediately mixed with five volumes of TRIzol reagent, and thoroughly homogenized. The samples were then transported on dry ice to a −80°C freezer for storage. The entire process was completed within 30 min to preserve RNA integrity.

### 2.2 Total RNA isolation

Total RNA was isolated from whole blood samples using the TRIzol^®^ reagent (Takara, Japan) following the manufacturer's protocol. The RNA concentration and purity were determined using a NanoDrop ND-2000 spectrophotometer (Thermo Scientific, USA), and the RNA integrity was evaluated through 1% agarose gel electrophoresis as well as with the Agilent 5400 Bioanalyzer system (Agilent Technologies, USA). All procedures, including centrifugation and plasma aliquoting, were conducted under temperature-controlled conditions (4°C) to maintain sample stability. The separated plasma was aliquoted into cryotubes and immediately frozen at −80°C to preserve integrity. Until further processing, all samples were stored in a biobank facility at −80°C, with temperature monitored continuously to ensure optimal conditions.

### 2.3 Library preparation for RNA sequencing

Total RNA with an RNA integrity number (RIN) ≥7.0, extracted from blood samples, was used for transcriptome library construction. The RNA sequencing libraries were prepared and sequenced by Beijing Novogene Bioinformatics Technology (http://www.novogene.com). Five micrograms of RNA from each peripheral blood sample served as the starting material for library preparation. RNA sequencing was conducted using next-generation high-throughput sequencing technology, with two distinct types of libraries constructed from the same sample to capture a broad spectrum of RNA species. These libraries included a ribosomal RNA-depleted strand-specific library for long RNA fragments, enabling the sequencing of lncRNA, circRNA, and mRNA, and a small RNA library for short RNA fragments, facilitating the sequencing of miRNA and other small RNAs.

### 2.4 Clustering and sequencing

Clustering of the indexed samples was performed using the TruSeq PE Cluster Kit v3-cBot-HS (Illumina) on the cBot Cluster Generation System, following the manufacturer's protocol. Post-cluster generation, libraries were sequenced on the Illumina HiSeq™ 4,000 platform (Illumina, San Diego, CA, USA) with 150 bp single-end reads. The raw sequencing data, obtained in FASTQ format, were processed using an in-house Perl script. To ensure the integrity of downstream analyses, the raw reads were subjected to a stringent filtering process. This included the removal of low-quality reads, adapter sequences, short reads, and sequences with a high rate of ambiguous bases (N). The filtered high-quality clean data were evaluated using quality metrics such as Q20, Q30, and GC content. Only data meeting these quality standards were retained for subsequent analyses.

### 2.5 Reference genome

The reference genome and gene model annotation files were downloaded from a recognized genomic database. The reference genome was indexed using Bowtie2 v2.2.8, and paired-end clean reads were aligned to the reference genome utilizing Bowtie2, ensuring high alignment accuracy (14).

### 2.6 Differential expression analysis

Differential expression analysis was performed using the DESeq R package (version 4.2.1), which implements statistical routines for assessing differential expression in digital gene expression (DGE) data based on a negative binomial distribution. To control for false discovery, *p*-values were adjusted using the Benjamini & Hochberg method. Genes with an adjusted *p*-value (q-value) < 0.05 were considered differentially expressed (DE). All analyses were conducted using the default parameters of DESeq to ensure consistency and reproducibility.

### 2.7 Bioinformatic analysis of differentially expressed mRNA

Gene Ontology (GO) enrichment analysis was performed on the differentially expressed (DE) mRNA using the GOseq R package to predict gene functions. These functions were categorized into three primary subgroups: Biological Process (BP), Molecular Function (MF), and Cellular Component (CC). The results of the GO enrichment analysis were visualized using DE mRNA GO Functional Enrichment bubble plot to represent the most enriched categories. Additionally, pathways associated with the DE mRNA were identified through the Kyoto Encyclopedia of Genes and Genomes (KEGG) pathway analysis.

Target genes of significantly differentially expressed miRNAs were predicted using two bioinformatics tools: miRanda and RNAhybrid. The intersection of significantly differentially expressed miRNAs and mRNAs was determined based on the principle of miRNA-mediated mRNA inhibition. Consequently, significantly downregulated miRNAs paired with significantly upregulated mRNAs, and significantly upregulated miRNAs paired with significantly downregulated mRNAs, were identified as potential regulatory pairs.

To explore the regulation of target gene mRNA expression by lncRNA through co-localization or co-expression, we performed an intersection analysis between the target genes of differentially expressed lncRNAs and differentially expressed mRNAs. When the target genes of differentially expressed lncRNAs were also identified as significantly differentially expressed mRNAs across samples, it suggested a higher likelihood of direct or indirect regulatory effects of the lncRNA on the DE mRNA. The union of target genes influenced by both co-localization and co-expression with DE mRNAs was selected for further analysis. The prediction of miRNAs targeting lncRNAs was also conducted using the miRanda software. Finally, Cytoscape software was employed to construct a regulatory interaction network diagram illustrating lncRNA-miRNA-mRNA interactions.

### 2.8 qPCR validation of differential RNA

To validate the differentially expressed (DE) RNAs identified by RNA-seq, quantitative real-time PCR (qRT-PCR) was performed using six total RNA samples. A total of nine DE RNAs were selected for validation. For the analysis of lncRNAs and mRNAs, reaction mixtures were prepared following the instructions provided with the PrimeScript™ RT reagent Kit, which includes gDNA Eraser. Complementary DNA (cDNA) templates were synthesized from 2 μg of total RNA. qRT-PCR amplification was conducted using TB Green™ Premix Ex Taq™ II (Takara, Japan) on an ABI 7500 real-time PCR system (Applied Biosystems, USA). The optimal reaction conditions were set as follows: initial denaturation at 95°C for 30 seconds, followed by 40 cycles of denaturation at 95°C for 5 seconds, and annealing/extension at 60°C for 30 seconds. The specificity of the amplification was confirmed by a single peak in the melting curve analysis, indicating accurate and specific amplification. The verification analysis of miRNAs were performed according to the instructions of the miRNA reverse transcription kit (Takara, Japan). All primers used for the RNA analysis were designed and synthesized by Genesis Biotechnology (Nanning, China). Actin was employed as an internal control, and the relative gene expression levels were calculated using the 2–ΔΔCt method.

### 2.9 Statistical analysis

All statistical analyses were conducted using SPSS software (version 26.0). Graphical representations were created using GraphPad Prism software (version 9.5) and Cytoscape (version 3.10.0). Image processing were performed with Adobe Photoshop (version 25.0.0) and sangerbox online tool (http://sangerbox.com/). For comparisons between two independent groups, Student's *t*-test was employed. Data are presented as mean ± standard error (SE). Each experiment was conducted in triplicate to ensure reproducibility. A *p*-value of < 0.05 was considered statistically significant.

## 3 Results

### 3.1 Clinical information of the study population

In the experimental group (aSAH-MI), the initial cardiac troponin I (cTnI) levels of all six samples exceeded the 99th percentile upper reference limit (0.033 ng/ml), with values of 1.045, 0.638, 0.278, 0.211, 0.203, and 0.058 ng/ml, respectively. Subsequent re-examinations showed a decrease in cTnI levels to 0.170, 0.365, 0.236, 0.037, 0.069, and 0.002 ng/ml, respectively, corresponding to reductions of 0.875, 0.273, 0.042, 0.174, 0.134, and 0.056 ng/ml. The experimental group (aSAH-MI) meets the diagnostic criteria for myocardial injury. In contrast, all six samples in the control group (aSAH-nonMI) had cTnI levels below the 99th percentile upper reference limit (0.033 ng/ml), with values of 0.015, 0.010, 0.007, 0.003, 0.002, and 0.000 ng/ml, respectively, indicating that the control group (aSAH-nonMI) does not meet the diagnostic criteria for myocardial injury. There were no statistically significant differences in baseline characteristics between the two groups, including age, sex, hypertension, and diabetes (all *P* > 0.05; see Table 1), indicating comparability of the groups. Table 1 presents key clinical information for both the experimental and control groups.

**Table 1:** Clinical information of the study population.

**Variables**	**MI**	**Control**	***p*-value**
Age (years)	60(47, 62.75)	41.75(41.75, 61.25)	0.334
Male gender (*n* (%))	2(33.33%)	2(33.33%)	1
History of heart disease (*n* (%))	0(0)	0(0)	1
Hypertension (*n* (%))	4(66.7%)	3(50%)	0.575
Diabetes (*n* (%))	1(16.7%)	0(0)	0.317
Neoplasm (*n* (%))	0(0)	0(0)	1
Acute respiratory failure (*n* (%))	0(0)	0(0)	1
Uncontrolled metabolic disorders (*n* (%))	0(0)	0(0)	1
Immune system diseases (*n* (%))	0(0)	0(0)	1
Severe infections (*n* (%))	0(0)	0(0)	1
Current smoking (*n* (%))	0(0)	1(16.7%)	0.317
Alcohol intake (*n* (%))	0(0)	1(16.7%)	0.317
Liver dysfunction (AST or ALT≥120U/L) (*n* (%))	0(0)	0(0)	1
Renal failure (creatine≥120μmol/L) (*n* (%))	0(0)	0(0)	1
LVEF < 50% (*n* (%))	0(0)	0(0)	1
Arrhythmology (tachycardia or bradycardia) (*n* (%))	2(33.33%)	2(33.33%)	1
GCS (points)	14(12.25, 15)	15(11.25, 15)	0.432
Hunt-Hess grades≥III ratio (*n* (%))	3(50%)	2(33.33%)	0.575
WFNS grades≥III ratio (*n* (%))	3(50%)	2(33.33%)	0.575
Aneurysm location			
ICA (*n* (%))	3(50%)	2(33.33%)	0.575
MCA (*n* (%))	2(33.33%)	2(33.33%)	1
ACA (*n* (%))	1(16.7%)	0(0)	0.317
AComA (*n* (%))	0(0)	2(33.33%)	0.138

AST/ALT, Aspartate aminotransferase/Alanine aminotransferase; LVEF, Left ventricular ejection fraction; GCS, Glasgow coma scale; WFNS, World Federation of Neurosurgical Societies; ICA, Internal Carotid Artery, MCA, middle cerebral artery; ACA, arteriae cerebral anterior; AComA, anterior communicating artery.

### 3.2 Expression profiles of lncRNA, miRNA, and mRNA in aSAH-MI patients

**Figure 1:**
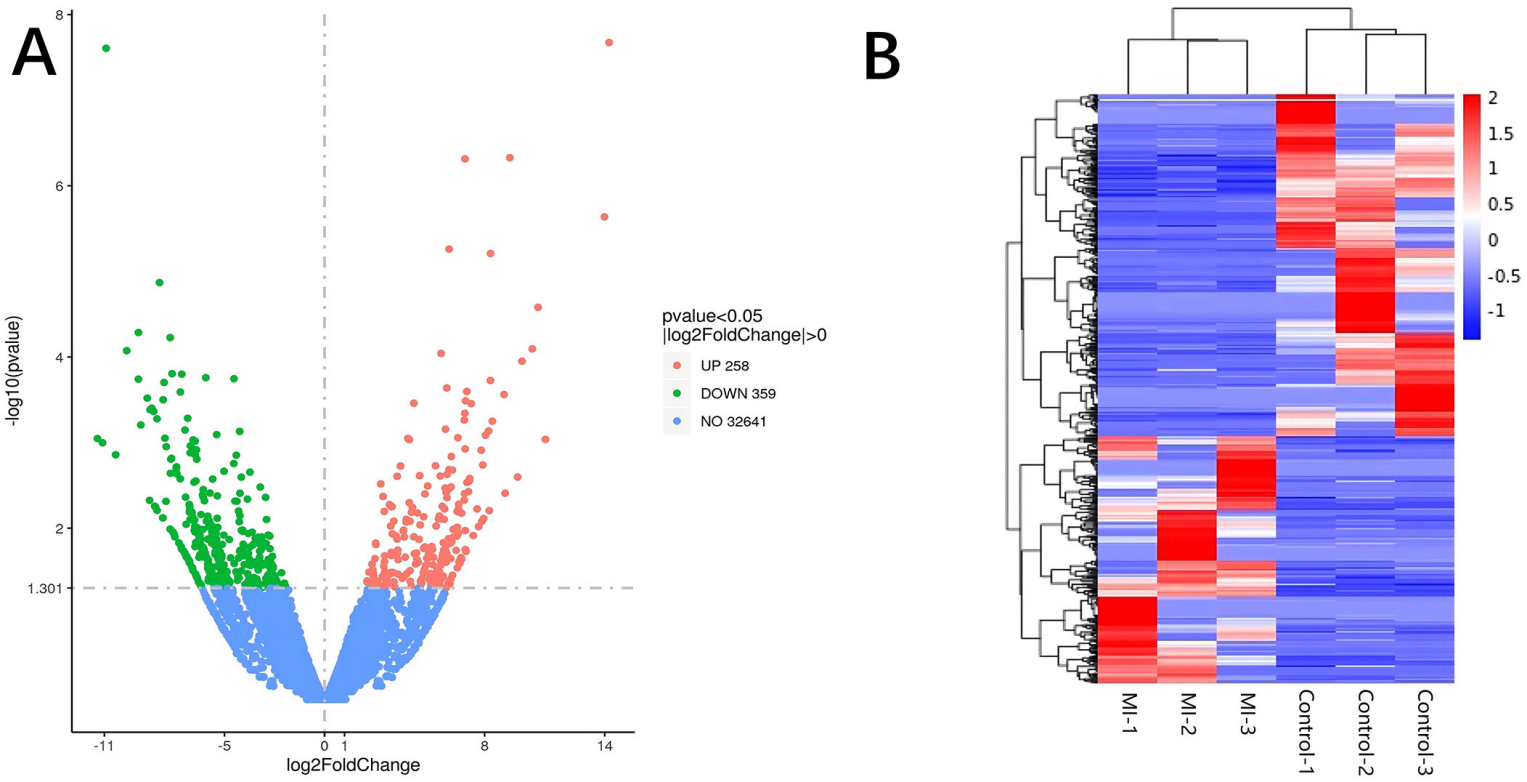
DE lncRNA volcano plot **(A)** and clustering heatmap **(B)**.

**Figure 2:**
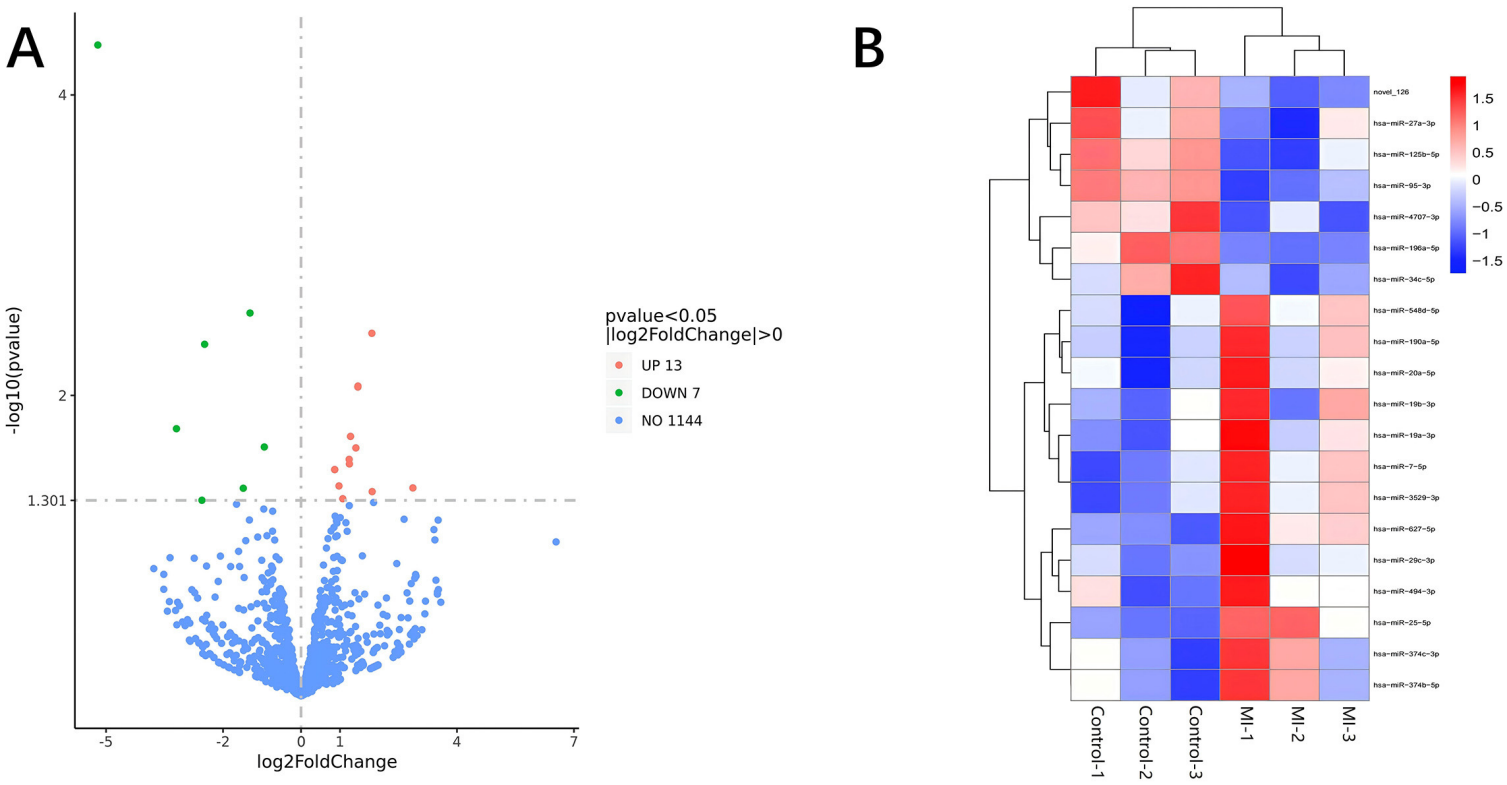
DE miRNA volcano plot **(A)** and clustering heatmap **(B)**.

**Figure 3:**
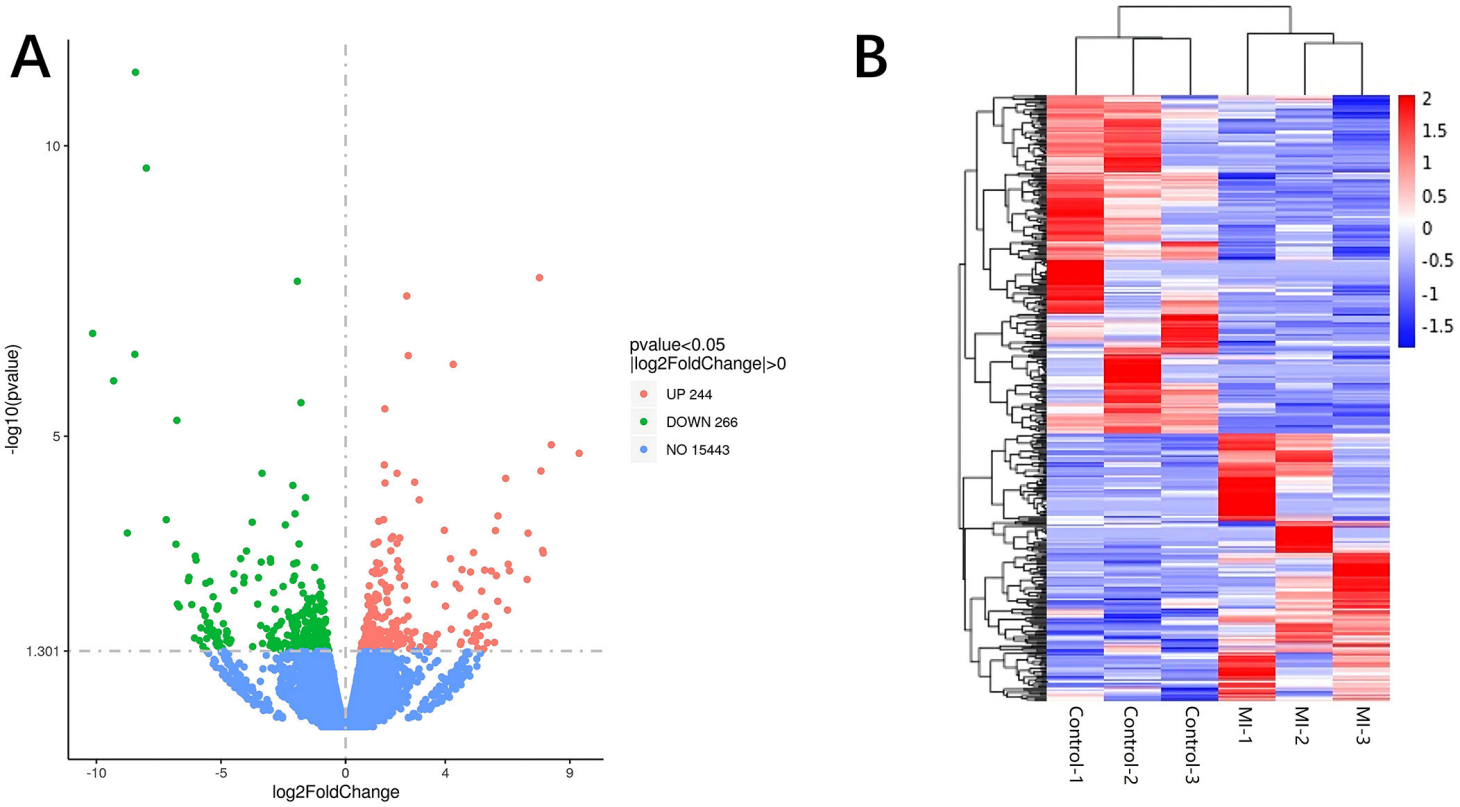
DE mRNA volcano plot **(A)** and clustering heatmap **(B)**.

Significant DE RNAs between aSAH-MI patients and controls were identified using a *p-*value threshold of < 0.05. A total of 617 lncRNAs, 20 miRNAs, and 510 mRNAs were found to be significantly differentially expressed. Among these, 258 lncRNAs, 13 miRNAs, and 244 mRNAs were significantly upregulated, while 359 lncRNAs, 7 miRNAs, and 266 mRNAs were significantly downregulated in aSAH-MI patients compared to controls. Volcano plots visualized the significant DE RNAs in aSAH-MI, with red dots representing significantly upregulated RNAs and green dots representing downregulated RNAs (Figures 1A, 2A, 3A). Hierarchical clustering analysis revealed that the RNA expression patterns of aSAH-MI patients were distinguishable from those of the control group (Figures 1B, 2B, 3B).

### 3.3 GO and KEGG analyses of mRNA

**Figure 4:**
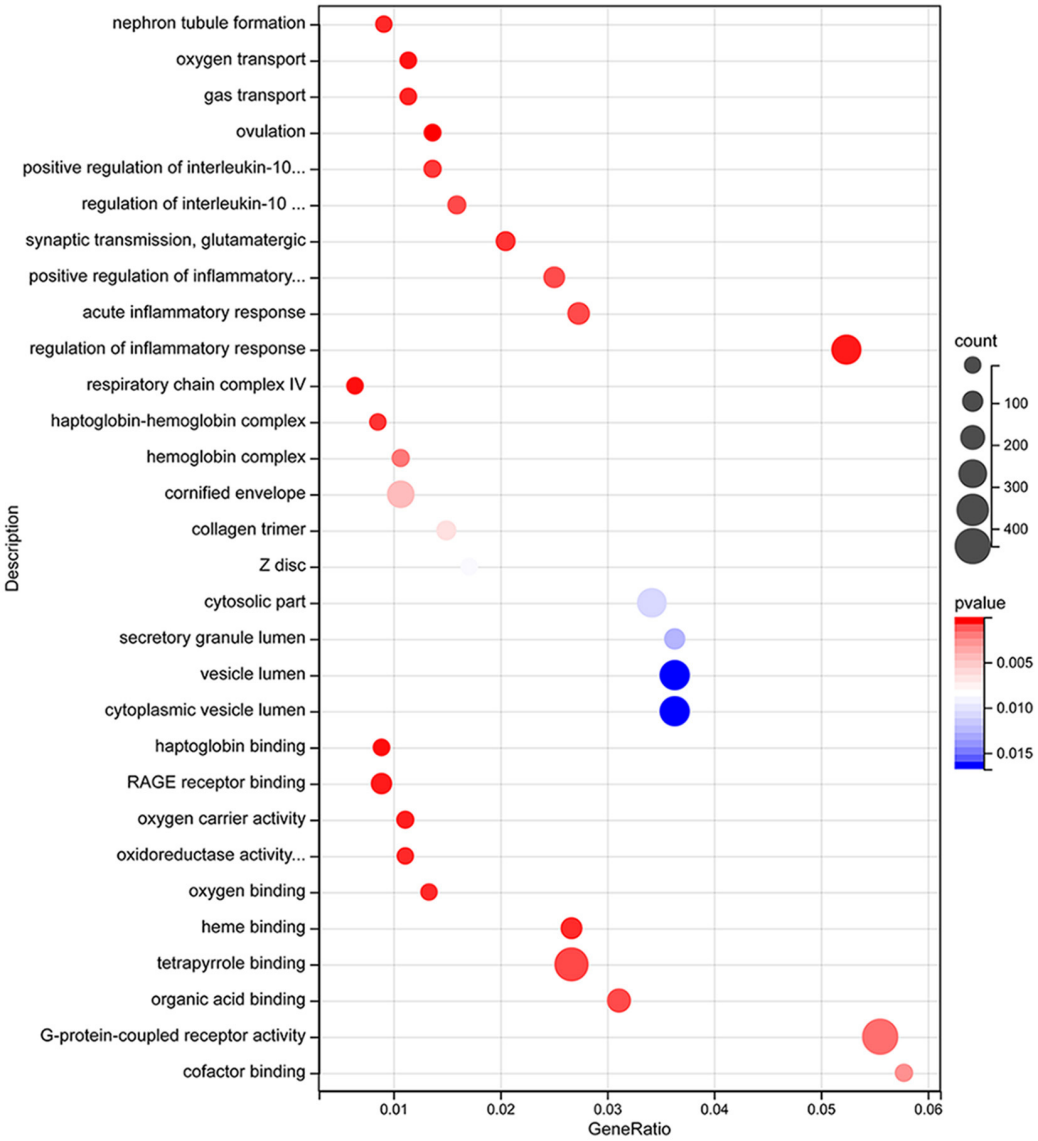
DE mRNA GO functional enrichment bubble plot.

Differentially expressed (DE) mRNAs identified in aSAH-MI patients were subjected to Gene Ontology (GO) annotation and enrichment analysis, as well as Kyoto Encyclopedia of Genes and Genomes (KEGG) pathway analysis, to elucidate their potential biological roles. GO analysis categorized the DE mRNAs into three main classes: Biological Process (BP), Cellular Component (CC), and Molecular Function (MF). The analysis revealed that these mRNAs were significantly associated with a range of biological processes, including ion transport, immune regulation, and myocardial cell contraction. Regarding cellular components, the dysregulated mRNAs were predominantly localized to cytosolic parts, intracellular organelles, protein complexes, and muscle fibers. The molecular functions of these mRNAs were primarily involved in oxygen carrier activity, oxygen binding, oxidoreductase activity, ion channel activity, protein binding, cytokine binding, and enzyme activity (Figure 4). These findings suggest significant alterations in cellular and molecular functions, as well as peripheral blood metabolism, in aSAH-MI patients compared to controls. Such changes are closely linked to the pathological mechanisms underlying brain injury.

**Figure 5:**
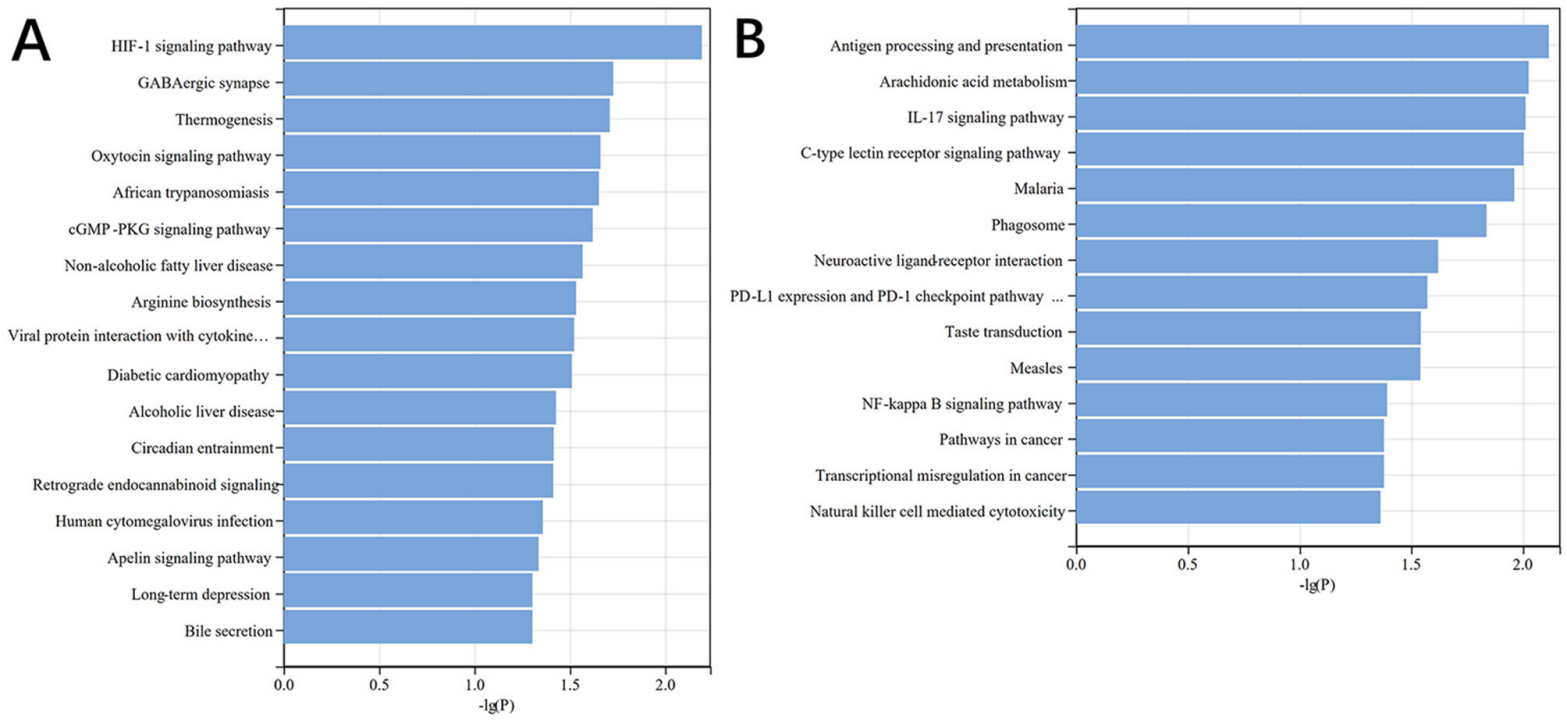
DE mRNA KEGG histogram. **(A)** KEGG pathways associated with upregulated genes, **(B)** KEGG pathways associated with downregulated genes.

Subsequent KEGG pathway enrichment analysis further highlighted the involvement of DE mRNAs in various signaling pathways, particularly those related to vascular dilation and constriction, neurotransmission, and the regulation of cardiac function (Figure 5). These pathways, which include those regulating endothelial function and neurotransmitter release, are critical in maintaining cardiovascular homeostasis and could play significant roles in the pathophysiology of myocardial injury following aSAH.

### 3.4 Construction and analysis of the lncRNA-miRNA-mRNA network

**Figure 6:**
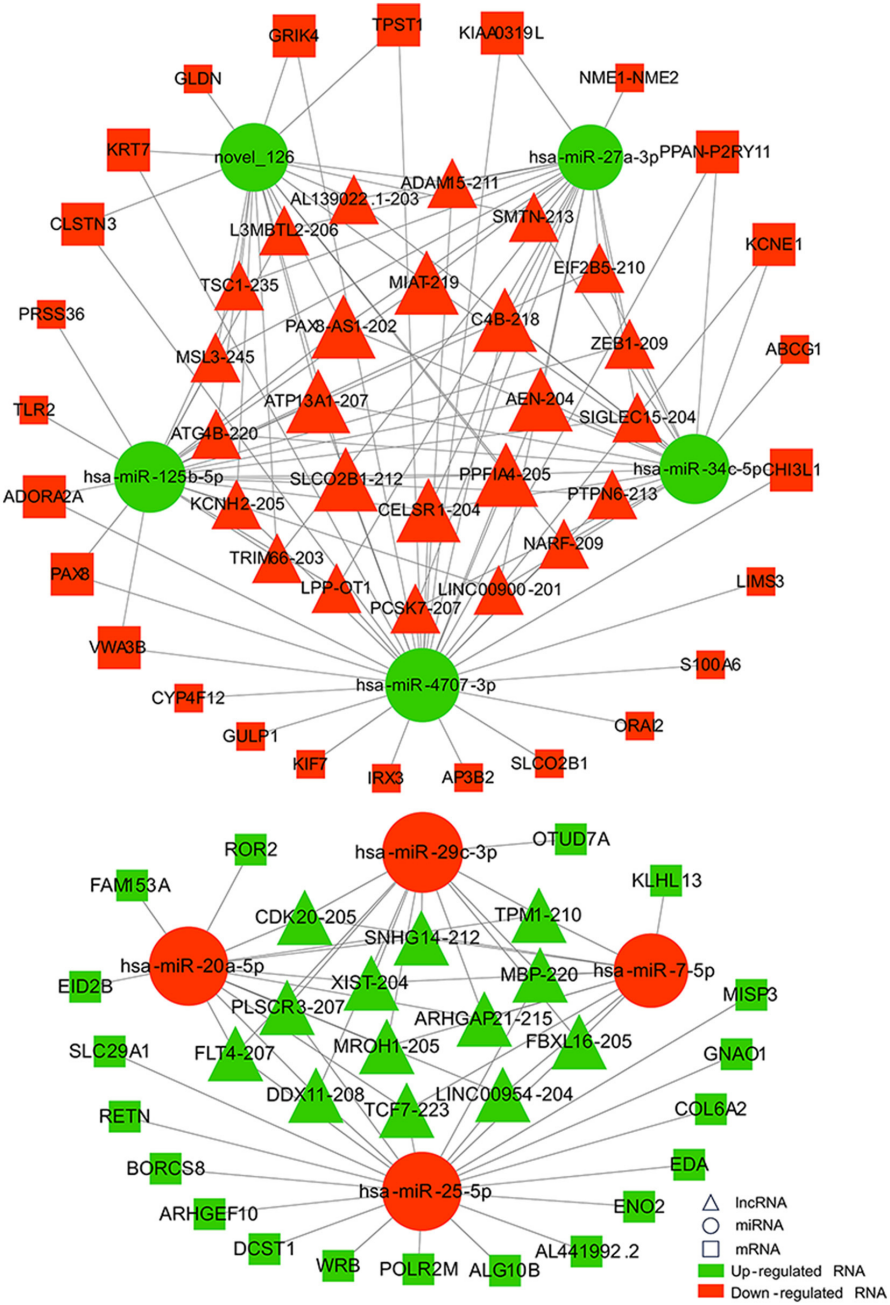
Network diagram of lncRNA-miRNA-mRNA interactions. Triangles represent lncRNAs, circles represent miRNAs, and squares represent mRNAs. Red indicates up-regulated RNAs, and green indicates down-regulated RNAs.

**Figure 7:**
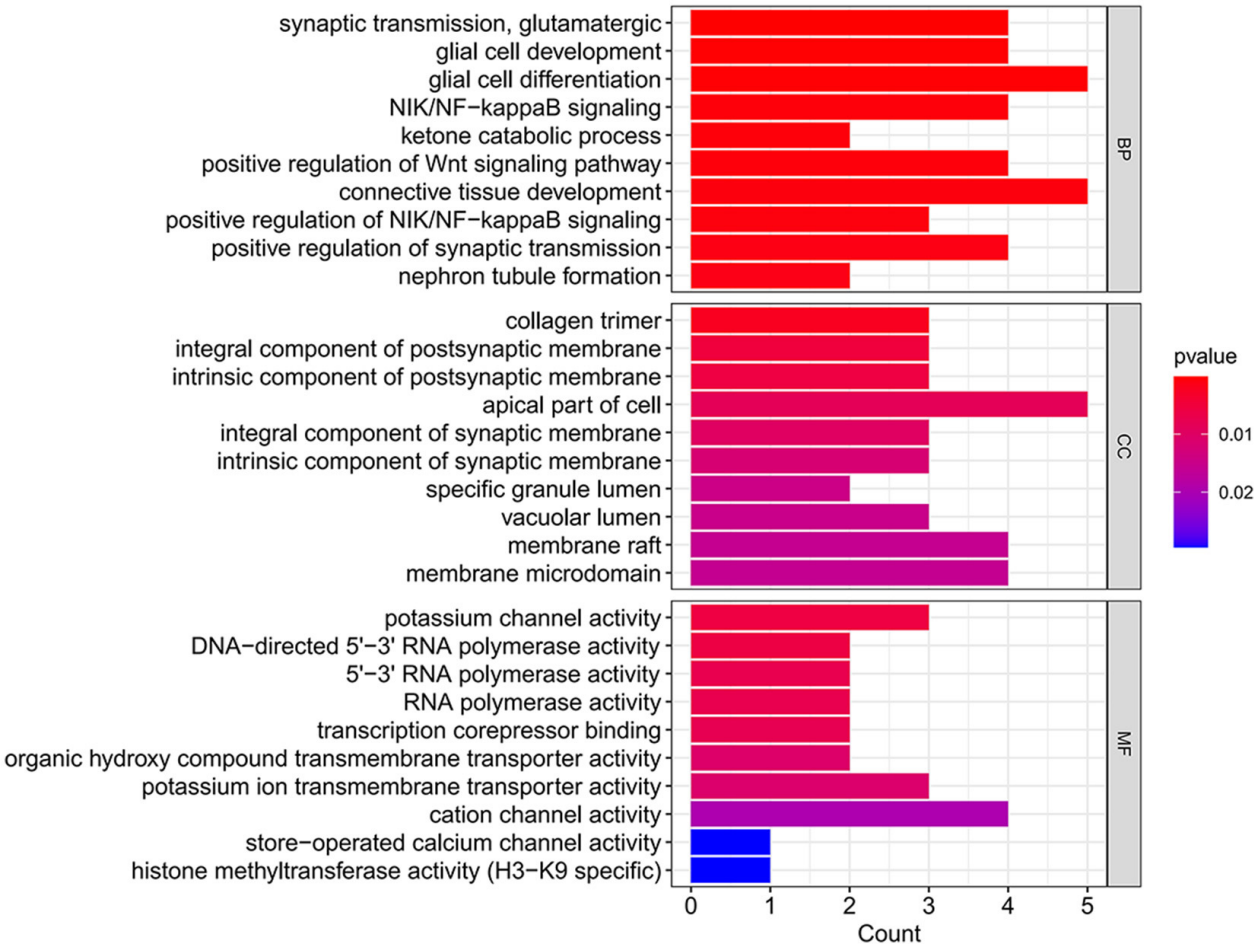
GO analysis histogram of DE mRNAs in the ceRNA network.

**Figure 8:**
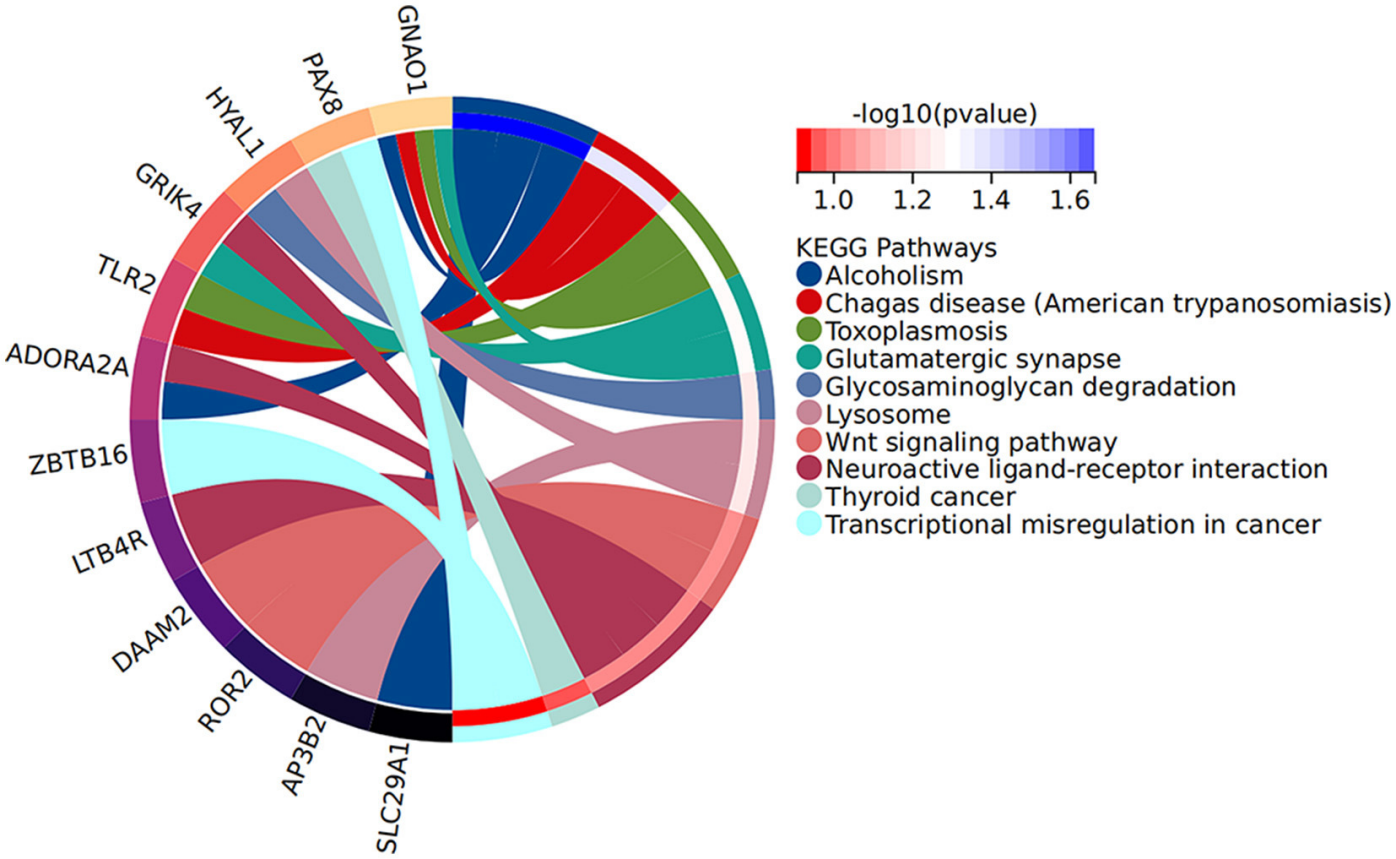
KEGG pathway histogram of DE mRNAs in the ceRNA network.

Increasing evidence supports the role of long non-coding RNAs (lncRNAs) as miRNA sponges, regulators of splicing and transcription, and modulators of host gene expression through the sequestration of specific miRNAs. In this study, we utilized the miRanda and RNAhybrid databases to predict miRNA target genes. The intersection of predictions from both databases, combined with the significantly differentially expressed mRNAs identified through RNA sequencing, was selected as the final set of target mRNAs. By analyzing sequence complementarity between competing endogenous RNAs (ceRNAs), the negative correlation in their expression levels, and the regulatory relationships among lncRNAs, miRNAs, and mRNAs, we constructed an lncRNA-miRNA-mRNA network. This network encompassed 287 lncRNAs, 9 miRNAs, and 63 mRNAs, forming 2861 ceRNA interactions (Figure 6). Within the network, the lncRNAs exhibiting the highest degree of connectivity were CELSR1-204, SLCO2B1-212, AEN-204, PPFIA4-205, and MIAT-219. The miRNA with the greatest connectivity was hsa-miR-4707-3p, while the mRNAs with the highest connectivity included CHI3L1, ADORA2A, PAX8, VWA3B, and KCNE1. Further functional analysis of the mRNAs within this network revealed their involvement in key biological processes, including glial cell development and activation, cardiac conduction, and the regulation of NF-κB signaling (Figure 7). Additionally, these genes were linked to critical signaling pathways such as the Wnt signaling pathway (Figure 8).

### 3.5 Validation of selected RNA expression levels

**Figure 9:**
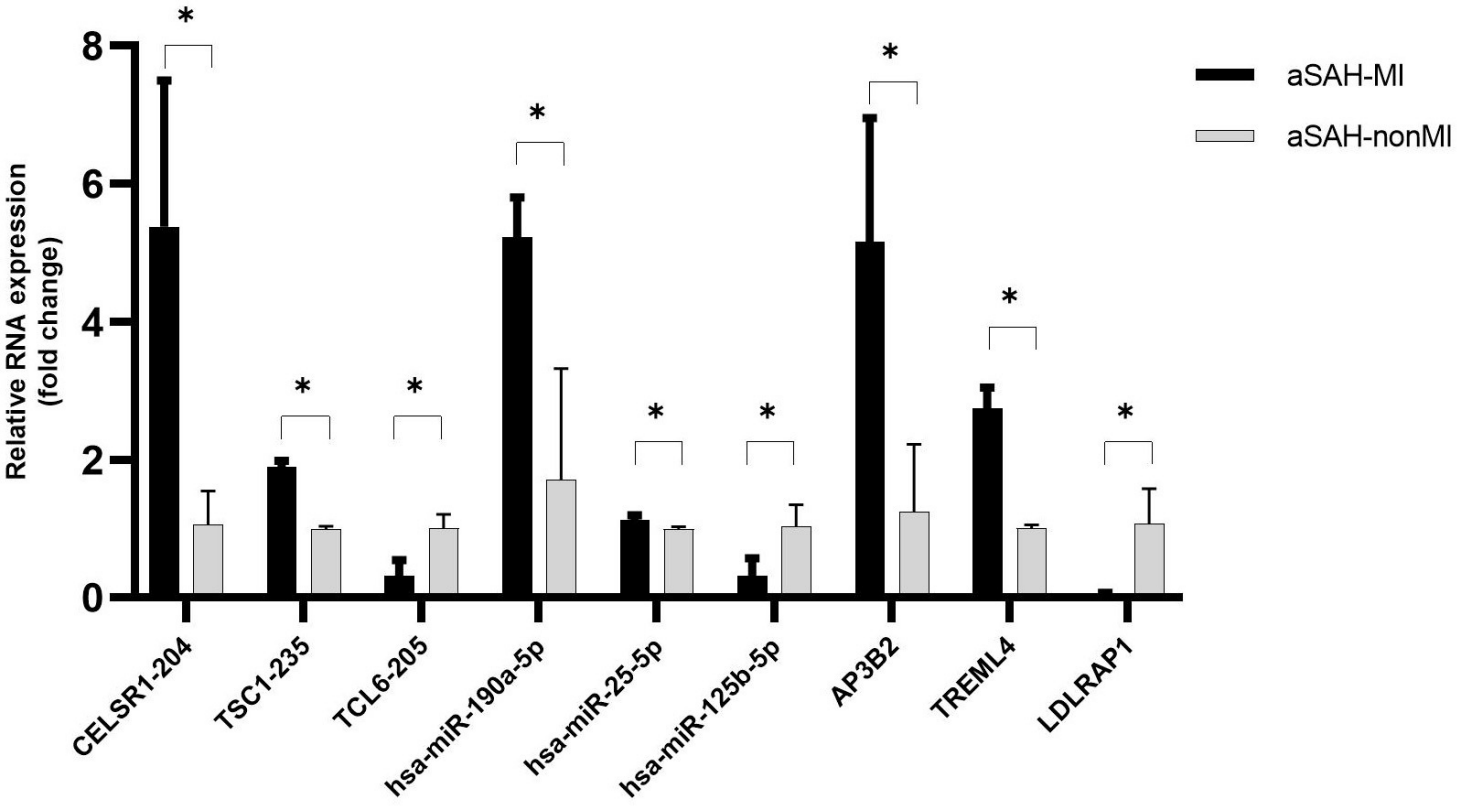
qRT-PCR validation results of DE RNAs. * indicates *P* < 0.05. The experiment was performed in three biological replicates, and the samples used for PCR validation were different from those used for transcriptome sequencing.

To confirm the accuracy of the RNA-Seq data, two upregulated and one downregulated RNA were selected from each of the three classes of differentially expressed (DE) RNAs (lncRNAs, miRNAs, and mRNAs). Quantitative real-time PCR (qRT-PCR) analysis was performed to assess the expression levels of these RNAs in three patients with aSAH-MI and three control subjects. The qRT-PCR results demonstrated expression patterns consistent with the RNA-Seq data, thereby confirming the reliability of the RNA-Seq findings. This validation reinforces the accuracy of the RNA-Seq analysis, providing robust support for the data's validity (Figure 9).

## 4 Discussion

In this study, we explored the expression profiles of long non-coding RNAs (lncRNAs) and messenger RNAs (mRNAs) associated with myocardial injury following aneurysmal subarachnoid hemorrhage (aSAH) using next-generation sequencing technology. A comprehensive analysis revealed 617 differentially expressed lncRNAs and 510 differentially expressed mRNAs, providing valuable insights into the molecular mechanisms underlying post-aSAH myocardial injury. To further elucidate the functional roles of these differentially expressed genes, we conducted extensive bioinformatics analyses, including Gene Ontology (GO) and Kyoto Encyclopedia of Genes and Genomes (KEGG) pathway analyses, as well as a competing endogenous RNA (ceRNA) network analysis.

The GO analysis identified 754 significantly enriched terms, highlighting the involvement of the differentially expressed mRNAs in key biological processes such as ion transport, immune regulation, myocardial cell contraction, and cardiac conduction. KEGG pathway analysis further revealed 31 significantly enriched pathways, implicating signal transduction processes related to vascular tone, neurotransmission, and cardiac function, including the HIF-1, Apelin, cGMP-PKG, IL-17, and NF-κB signaling pathways. Notably, the cGMP-PKG signaling pathway, which mediates interactions between cyclic guanosine monophosphate (cGMP) and protein kinase G (PKG), plays a crucial role in cardiac hypertrophy and heart failure management (15, 16). Meanwhile, NF-κB signaling pathway, as a crucial cellular signaling cascade, participates in various biological processes including immune response and apoptosis. Previous studies have identified NF-κB as a key mediator in aneurysm formation (17). Multiple genes mediate early brain injury and neuroinflammation following subarachnoid hemorrhage through the NF-κB signaling pathway (18, 19). The observed downregulation of the cGMP-PKG pathway and upregulation of the NF-κB pathway in this study underscore their potential roles in the pathogenesis of myocardial injury following aSAH. Future studies should focus on experimentally validating the involvement of these signaling pathways in this context.

LncRNAs are increasingly recognized for their ability to function as miRNA sponges, thereby modulating mRNA expression by sequestering specific miRNAs (20–22). The intricate interactions between mRNAs and ncRNAs were further explored through the construction of a ceRNA network in this study, which included 287 lncRNAs, 9 miRNAs, and 63 mRNAs, forming a total of 2,861 lncRNA-miRNA-mRNA interactions. This network suggests that differentially expressed lncRNAs may regulate gene transcription by acting as miRNA sponges, contributing to the development of myocardial injury after aSAH.

In the ceRNA network we constructed, CHI3L1 is one of the mRNAs with the highest degree, and its expression is upregulated in this study. CHI3L1 (Chitinase-3-like protein 1) is a gene encoding a secretory glycoprotein, and its encoded protein, also known as YKL-40, can be synthesized and secreted by astrocytes (23). Genome-wide association studies have shown that elevated levels of YKL-40 are associated with an increased risk of large artery stroke (24). In a prospective study involving 10,472 stroke patients, high levels of YKL-40 were also observed to be independently associated with recurrent stroke and adverse functional outcomes (25). Another study involving 4,298 patients with coronary artery disease reported that elevated serum YKL-40 levels were associated with more severe coronary artery disease, and higher YKL-40 levels were independent predictors of overall mortality and cardiovascular mortality (26). In this study, upregulation of CHI3L1 was observed, and its role in myocardial injury after aSAH is not yet clear. Subsequent investigations, such as CHI3L1 knockout/overexpression, may help elucidate the role of CHI3L1.

Further analysis of the mRNAs within the ceRNA network indicated their involvement in critical biological processes, including astrocyte development and activation, cardiac conduction, and regulation of the NF-κB signaling pathway. Additionally, these genes were implicated in key signaling pathways, such as the Wnt signaling pathway and Toll-like receptor signaling pathway. The concordance between the functions of the significantly differentially expressed mRNAs and those within the network suggests that these genes may serve as crucial regulators of myocardial injury following aSAH.

A limitation of this study is that it is confined to transcriptomic-level research. Emerging evidence suggests that metabolomics is increasingly valuable in revealing the pathophysiological processes underlying aSAH, it has been used to identify changes in glucose, lipid, and protein metabolism, which are associated with the severity of aSAH and its complications, such as cardiac dysfunction (27). For instance, alterations in taurine and ornithine levels are closely related to functional outcomes (27). Metabolomics can complement transcriptomic studies, offering a holistic view of the molecular and metabolic disturbances in aSAH. Future integrative research is expected to bridge the gap between gene expression changes and physiological responses.

In summary, this study has identified and characterized the expression profiles of lncRNAs and mRNAs associated with myocardial injury after aSAH. Our findings provide a foundation for further exploration into the biological functions and regulatory mechanisms of these differentially expressed genes, offering potential avenues for therapeutic intervention in post-aSAH cardiac complications.

## 5 Conclusions

This study successfully constructed the RNA expression profile in the blood of patients with myocardial injury following aSAH using transcriptome sequencing and identified significantly differentially expressed miRNAs, mRNAs, and lncRNAs. Through bioinformatic analyses, including GO, KEGG, miRNA-mRNA network analysis, and lncRNA-miRNA-mRNA network analysis, we demonstrated that these differentially expressed genes are likely to play significant roles in the pathogenesis of myocardial injury following aSAH.

## Data Availability

The data presented in the study are deposited in the Genome Sequence Archive for Human. Small RNA Library, accession: HRA011828, https://bigd.big.ac.cn/gsa-human/browse/HRA011828 and Ribosome-depleted Strand-specific RNA Library, accession: HRA011869, https://bigd.big.ac.cn/gsa-human/browse/HRA011869.
