# Supplementary material for: Transcriptomic profiling and bioinformatic insights into myocardial injury following aneurysmal subarachnoid hemorrhage
Source: Front Neurol. 2025 Jun 24;16:1492398. doi: 10.3389/fneur.2025.1492398 (PMC12235911; doi:10.3389/fneur.2025.1492398)
Supplement: Supplementary file 1 [file Table_1.docx]

Table S1.qRT-PCR primers used

| RNA Type | RNA Primers | Sequences |
| --- | --- | --- |
| miRNA | hsa-miR-125b-5p | CCGTCCCTGAGACCCTAACTTGT |
| miRNA | hsa-miR-25-5p | GGAGGCGGAGACTTGGGCAATT |
| miRNA | hsa-miR-190a-5p | GGGTGGGTCTGATATGTTTGATATATTAGGT |
| lncRNA | TSC1-235 | Forward primer TGTCCCACCTGATCTGTCAC |
|  |  | Reverse primer AGGCAGGCCAAAACCAACT |
| lncRNA | CELSR1-204 | Forward primer CCATCACAGTCAAGAGCCCTG |
|  |  | Reverse primer GACTCAGGACAAGGGGGAAGT |
| lncRNA | TCL6-205 | Forward primer TAAGGGGTTTGAGGATGTGGC |
|  |  | Reverse primer CCTATTTCAGGGTTTCTATCTCTGTC |
| mRNA | AP3B2 | Forward primer ACGTCTCCATTCAGCCACCT |
|  |  | Reverse primer TGGCAGCATGAGTTTCTCTGT |
| mRNA | TREML4 | Forward primer CCCAACCACGTCTCCTATGTG |
|  |  | Reverse primer TCCACATAGCACCAAGACCAG |
| mRNA | LDLRAP1 | Forward primer ATCGTGGCTACAGCTAAGGC |
|  |  | Reverse primer ATGCAAACACCTTGTCGTGC |
